# IGF2BP3‐dependent N6‐methyladenosine modification of USP49 promotes carboplatin resistance in retinoblastoma by enhancing autophagy via regulating the stabilization of SIRT1

**DOI:** 10.1002/kjm2.12902

**Published:** 2024-11-04

**Authors:** Lei Li, Ning Yang, Jian‐Hong Sun, Li‐Juan Wei, Yuan Gao

**Affiliations:** ^1^ Department of Ophthalmology Hainan General Hospital (Hainan Affiliated Hospital of Hainan Medical University) Haikou China

**Keywords:** autophagy, carboplatin resistance, IGF2BP3, N6‐methyladenosine

## Abstract

Retinoblastoma (RB) poses significant challenges in clinical management due to the emergence of resistance to conventional chemotherapeutic agents, particularly carboplatin (CBP). In this study, we investigated the molecular mechanisms underlying CBP resistance in RB, with a focus on the role of autophagy and the influence of ubiquitin‐specific peptidase 49 (USP49). We observed upregulation of USP49 in RB tissues and cell lines, correlating with disease progression. Functional assays revealed that USP49 promoted aggressive proliferation and conferred CBP resistance in RB cells. Furthermore, USP49 accelerated tumor growth and induced CBP resistance in vivo. Mechanistically, we found that USP49 facilitated CBP resistance by promoting autophagy activation. In addition, we identified insulin‐like growth factor 2 mRNA‐binding protein 3 (IGF2BP3)‐mediated N6‐methyladenosine (m^6^A) modification of USP49 as a regulatory mechanism, wherein IGF2BP3 upregulated USP49 expression in an m^6^A‐dependent manner. Moreover, USP49 stabilized SIRT1, a protein associated with CBP resistance and autophagy, by inhibiting its ubiquitination and degradation. Rescue experiments confirmed the pivotal role of SIRT1 in USP49‐mediated CBP resistance. Our findings delineate a novel molecular network involving USP49‐mediated autophagy in promoting CBP resistance in RB, offering potential targets for therapeutic intervention to enhance treatment efficacy and improve outcomes for RB patients.

## 1 INTRODUCTION

Retinoblastoma (RB), a malignancy arising from the developing retina, primarily afflicts infants and young children and severely compromises the vision and overall health of affected individuals.^1^ Despite a range of treatment modalities spanning from focal therapies to systemic chemotherapy, the emergence of resistance to conventional agents remains a significant impediment in clinical RB management.^2^ Carboplatin (CBP), a platinum‐based chemotherapeutic agent, has become integral to RB treatment.^3^ Nevertheless, the development of resistance to CBP markedly diminishes its efficacy and represents a critical hurdle in RB therapy,^4^ underscoring the imperative for deeper insights into the molecular mechanisms governing CBP resistance.

Chemoresistance is a multifaceted issue in which cancer cells acquire the ability to resist the cytotoxic effects of chemotherapy agents, making treatment less effective or even ineffective.^5^ In recent years, the intricate interplay between cellular mechanisms and chemoresistance has garnered substantial attention in cancer research.^6^ Autophagy, a fundamental cellular process involved in the degradation and recycling of cellular components,^7^ has emerged as a critical factor in modulating resistance to cancer treatment in human cancers by promoting cell survival during stress, inhibiting cell apoptosis, enhancing drug resistance to chemotherapy agents, and facilitating metabolic adaptation.^8^, ^9^ Consequently, targeting pro‐survival autophagy in conjunction with traditional therapies is being explored as a strategy to overcome resistance and improve treatment efficacy.^10^ Recent evidence indicates crosstalk between autophagy and various cellular processes, including cell proliferation, invasion, and apoptosis, which complicates the dynamics of chemoresistance in RB.^11^, ^12^ In addition, the inhibition of autophagy has been shown to enhance the sensitivity of RB cells to chemotherapy agents.^13^, ^14^ Prior research has established a link between dysregulated autophagy and aggressive tumor phenotypes, as well as resistance to CBP in RB.^15^ Mechanistically, autophagy can mitigate the cytotoxic effects of CBP by facilitating the degradation of damaged cellular components and promoting the survival of cancer cells.^16^ However, the precise molecular mechanisms linking autophagy to CBP resistance in RB remain elusive.

Ubiquitin‐specific peptidase 49 (USP49) has emerged as a crucial player in human cancers, exerting multifaceted roles in tumorigenesis.^17^ As a member of the deubiquitinase family, USP49 modulates protein stability and cellular signaling pathways by reversing ubiquitination, thereby regulating various cellular processes implicated in cancer progression.^18^ Notably, USP49 has garnered attention for its involvement in chemoresistance, wherein aberrant expression or activity of USP49 confers resistance to diverse chemotherapeutic agents in different cancer types.^19^, ^20^ Given the promoting role of USP49 in resistance to platinum‐based chemotherapeutic drugs in human cancers,^21^ USP49 may also participate in RB progression or CBP resistance in RB.

In this study, we delved into the intricate molecular mechanisms underlying CBP resistance in RB, particularly focusing on the involvement of autophagy in CBP resistance and elucidating how USP49 influences autophagic processes. In addition, we explored including the N6‐methyladenosine (m^6^A) modification of USP49 mediated by insulin‐like growth factor 2 mRNA‐binding protein 3 (IGF2BP3) and the impact of USP49 on the stabilization of sirtuin 1 (SIRT1). Through comprehensive elucidation of these molecular pathways, our study aims to provide novel insights into the mechanisms driving CBP resistance in RB, thereby paving the way for the development of targeted therapeutic strategies to overcome CBP resistance and improve treatment outcomes for patients with RB.

## 2 MATERIALS AND METHODS

### 2.1 Dataset analysis

Publicly available gene expression datasets were retrieved from the Gene Expression Omnibus (GEO) database. The GEO dataset GSE24673 was analyzed using GEO2R (https://www.ncbi.nlm.nih.gov/geo/geo2r/) to identify differentially expressed genes (DEGs) between RB tissues and normal samples.

### 2.2 Cell culture

Human embryonic kidney cell line (HEK293T), human retinal epithelial cell line (ARPE‐19), and RB cell lines (SO‐Rb 50, Y‐79, HXO‐RB44, and WERI‐Rb‐1) were obtained from the American Type Culture Collection (ATCC). ARPE‐19 cells and RB cell lines were cultured in RPMI‐1640 medium supplemented with 10% FBS (37°C; 5% CO_2_). HEK293T cells were cultured in DMEM‐H supplemented with 10% FBS in a humidified atmosphere (37°C; 5% CO_2_).

### 2.3 Establishment of CBP‐resistant RB cell line

As previously described,^22^ the CBP‐resistant RB cell line (Y‐79/CBP) was established by subjecting Y‐79 cells to progressively increasing concentrations of CBP over several months. Specifically, CBP was added when Y‐79 cells reached 70%–80% confluence. After a 2‐day exposure to CBP, the culture medium was replaced with fresh CBP‐free medium. Once the cells returned to normal growth, CBP was reintroduced. Finally, resistant cells were maintained in CBP at 5 μM. The resistance to CBP was confirmed by assessing the half‐maximal inhibitory concentration (IC50) value for CBP.

### 2.4 Plasmid construction, cell transfection, and lentivirus infection

Overexpression plasmids were constructed using the pcDNA3.1 vector. In brief, pcDNA3.1 empty vector (Vector), pcDNA3.1‐USP49 vector (USP49), pcDNA3.1‐SIRT1 vector (SIRT1), and pcDNA3.1‐IGF2BP3 vector (IGF2BP3) were synthesized by GenePharma (Shanghai, China). These plasmids were transfected into Y‐79 or Y‐79/CBP cells using lipofectamine 3000 (Invitrogen, USA). After 48 h, transfected cells were screened with G‐418 (200 μg/mL) to select stable clones.

Lentiviral production and cell infection were performed as previously described.^23^ Briefly, short hairpin RNA (shRNA) targeting USP49 (shUSP49#1: AGCTCACCAAACAGGTCTTAA; shUSP49#2: GCTCACCAAACAGGTCTTAAA) and IGF2BP3 (shIGF2BP3#1: GAAACTTCAGATACGAAATAT; shIGF2BP3#2: TCTGCGGCTTGTAAGTCTATT) and shRNA interference control (shNC: CCTAAGGTTAAGTCGCCCTCG) were cloned into pLKO.1 lentiviral vector (Sigma‐Aldrich) to construct corresponding silencing plasmids. HEK293T cells were plated into 6‐cm dishes (2 × 10^6^ cells) and co‐transfected with the pLKO.1 plasmid and packaging plasmids (psPAX2 and pMD2.G). After incubation for 48 h, lentiviral supernatants were collected, filtered, and concentrated to harvest lentiviruses. Y‐79 and Y‐79/CBP cells were infected with these lentiviruses, respectively. After 48 h, stable clones were selected with puromycin (5 μg/mL). Knockdown efficiency was verified by RT‐qPCR or western blotting.

### 2.5 RT‐qPCR

Total RNA was extracted from cells using TRIzol reagent (Thermo Fisher Scientific) according to the manufacturer's instructions. cDNA was synthesized using the PrimeScript RT reagent kit (Takara). Then, qPCR was performed using SYBR Green Master Mix (Thermo Fisher Scientific) on a real‐time PCR system. Gene expression levels were normalized to GAPDH.

### 2.6 Western blotting

Total protein was extracted from cells using RIPA lysis buffer (Thermo Fisher Scientific). Protein concentration was determined using the BCA protein assay kit. Equal amounts of protein were separated by 10% SDS‐PAGE and transferred onto PVDF membranes. Membranes were incubated with primary antibodies overnight at 4°C followed by secondary antibodies. Protein bands were visualized using an ECL detection system (Bio‐Rad Laboratories).

### 2.7 CCK‐8 assay

The IC50 values of CBP were determined using CCK‐8 assay (Dojindo). Briefly, cells were seeded into 96‐well plates at a density of 5000 cells per well and treated with varying concentrations of CBP (0, 5, 10, 25, 50, 100, 200 μM) for 48 h. CCK‐8 solution was added to each well, and absorbance was measured at 450 nm using a microplate reader. IC50 values were calculated using dose–response curves generated by nonlinear regression analysis.

### 2.8 Colony formation assay

For colony formation analysis, cells were seeded in 6‐well plates at a density of 500 cells per well and cultured for 7–10 days until visible colonies formed. Cells were then fixed with 4% paraformaldehyde and stained with crystal violet solution. Colonies containing ≥50 cells were counted under a microscope.

### 2.9 Flow cytometry

Cell apoptosis was assessed using flow cytometry with Annexin V‐FITC/PI staining. Cells were harvested, washed with PBS, and resuspended in a binding buffer. Annexin V‐FITC and propidium iodide (PI) were added to the cell suspension and incubated in the dark for 15 min at room temperature. Apoptotic cells were analyzed using a flow cytometer. Data analysis was performed using FlowJo software.

### 2.10 Animal study

The present animal study was approved by the Ethics Committee of The Affiliated Hospital of Qingdao University. Male BALB/c nude mice (6–8 weeks old) were obtained from Shanghai SLAC Laboratory and maintained in a room with a 12‐h light/dark cycle with free access to food and water. To evaluate tumor growth and CBP resistance in vivo, the xenograft tumor models were established. Mice were assigned to shNC, shNC+CBP, shUSP49#2, and shUSP49#2 + CBP groups (*n* = 6 mice/group). Briefly, each mouse received a subcutaneous injection of Y‐79/CBP cells transfected with shNC or shUSP49#2 (1 × 10^7^ cells) into the flank. A week later, CBP administration was initiated. Mice in each group received intraperitoneal injections of CBP (1 mg/kg) or equal volumes of PBS at the indicated time. The tumor volume was evaluated every 3 days (tumor volume = (length × width^2^)/2). After all mice were sacrificed, xenografted tumors were harvested for tumor weight measurement and immunohistochemical assessment.

### 2.11 Transmission electron microscopy (TEM)

For the observation of autophagosomes in vitro, RB cells were fixed with 2.5% glutaraldehyde in 0.1 M phosphate buffer (pH 7.4) for 2 h at room temperature. Following fixation, cells were washed with phosphate buffer and post‐fixed with 1% osmium tetroxide for 2 h. Samples were then dehydrated through a graded series of ethanol and embedded in epoxy resin. Ultrathin sections were cut using an ultramicrotome and mounted onto copper grids. Sections were stained with uranyl acetate and lead citrate. Autophagosomes were visualized specifically under the transmission electron microscope.

### 2.12 Co‐immunoprecipitation (Co‐IP)

Cells were lysed in IP lysis buffer (Thermo Fisher Scientific), and protein lysates were incubated with primary antibodies overnight at 4°C with rotation. Protein A/G beads (Santa Cruz Biotechnology, USA) were added to the lysates and incubated for an additional 2 h at 4°C. Beads were washed, and bound proteins were eluted by boiling in SDS sample buffer. Immunoprecipitated proteins were analyzed by western blotting.

### 2.13 Cycloheximide (CHX) chase experiment

To assess protein degradation rates, cells were treated with the protein synthesis inhibitor cycloheximide (CHX) at a concentration of 40 μg/mL. Cells were harvested at various time points (0, 4, 8, and 12 h) after CHX treatment. Total protein was extracted, and the levels of target proteins were analyzed by western blotting.

### 2.14 MeRIP–qPCR assay

MeRIP was performed using the Magna MeRIP m^6^A RNA Methylation Kit (MilliporeSigma). Briefly, total RNA was extracted from cells using TRIzol reagent and sheared into fragments using RNA fragmentation buffer. Fragmented RNA was immunoprecipitated using an m^6^A‐specific antibody conjugated to protein A/G magnetic beads. After elution, the methylated RNA was subjected to RT‐qPCR using gene‐specific primers.

### 2.15 RIP–qPCR

RIP was performed using the Magna RIP RNA‐Binding Protein Immunoprecipitation Kit (MilliporeSigma). Briefly, cells were lysed in RIP lysis buffer supplemented with RNase and protease inhibitors. Cell lysates were then incubated with protein A/G magnetic beads conjugated with antibodies specific to IGF2BP3. Following immunoprecipitation, RNA–protein complexes were eluted, and RNA was extracted. The enriched RNA was subjected to RT‐qPCR using gene‐specific primers.

### 2.16 Statistical analysis

All experiments were performed at least three times, and data are presented as mean ± standard deviation (SD). Statistical analysis was performed using GraphPad Prism software (GraphPad Software). Student's *t*‐test or one‐way analysis of variance (ANOVA) followed by Tukey's post hoc test was used for comparisons between groups. *p* < 0.05 was considered statistically significant.

## 3 RESULTS

### 3.1 Upregulation of USP49 in RB

**FIGURE 1:**
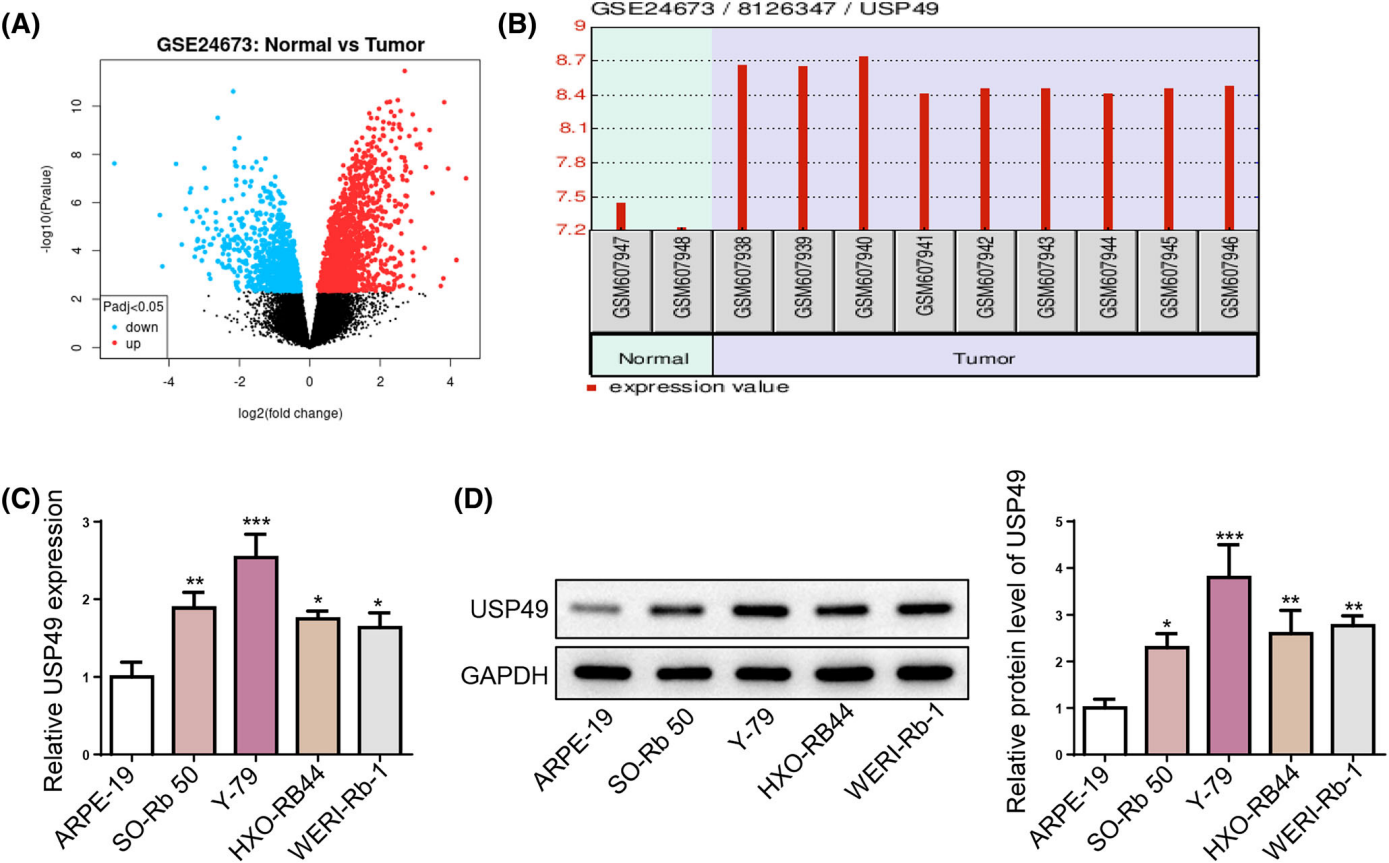
Upregulation of USP49 in RB. (A) Volcano plot for DEGs in RB. (B) USP49 expression in RB tissues compared with normal tissues. (C,D) USP49 mRNA and protein expression levels in a normal human retinal epithelial cell line (ARPE‐19) and 4 RB cell lines (SO‐Rb 50, Y‐79, HXO‐RB44, and WERI‐Rb‐1) were detected by RT‐qPCR and Western blotting. *p < 0.05; **p < 0.01.

To screen DEGs between RB and normal samples, the GEO dataset (GSE24673) was analyzed using GEO2R (Figure 1A). Among these DEGs, USP49 was upregulated in RB tissues compared with normal tissues (Figure 1B). Subsequent examination of USP49 mRNA and protein expression levels in normal and RB cell lines confirmed significant increases in both mRNA and protein levels in RB cell lines (Figure 1C,D). Collectively, these findings indicate an association between high USP49 expression and RB progression.

### 3.2 USP49 knockdown inhibits aggressive proliferation and CBP resistance in RB cells

**FIGURE 2:**
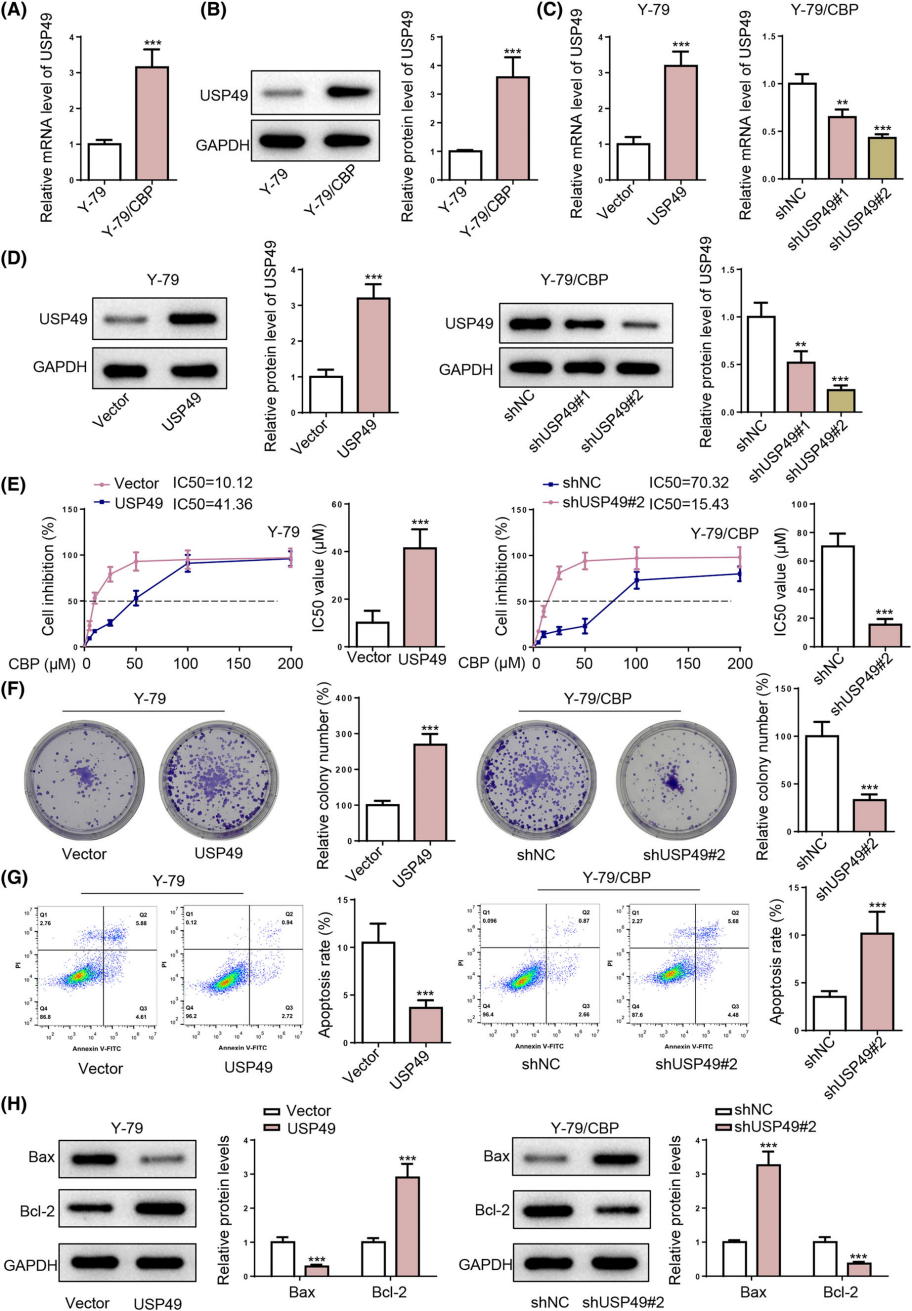
USP49 knockdown inhibits aggressive proliferation and CBP resistance in RB cells. (A,B) USP49 mRNA and protein expression levels in Y‐79 cells and Y‐79/CBP cells. Then, Y‐79 cells were transfected with vector or USP49; Y‐79/CBP cells were transfected with shNC, shUSP49#1, or shUSP49#2. (C,D) USP49 mRNA and protein expression levels in Y‐79 cells and Y‐79/CBP cells. (E) IC50 of CBP for Y‐79 cells and Y‐79/CBP cells from each group were detected using CCK‐8 assay. (F) Cell proliferation of Y‐79 cells and Y‐79/CBP cells from each group was evaluated by colony‐formation assay. (G) The apoptotic rates of Y‐79 cells and Y‐79/CBP cells from each group were assessed by flow cytometry. (H) Bax and bcl‐2 protein levels in Y‐79 cells and Y‐79/CBP cells from each group. *p < 0.05; **p < 0.01.

To investigate the impact of USP49 on CBP resistance in RB, we established a CBP‐resistant RB cell line (Y‐79/CBP) based on the RB cell line (Y‐79) first. Y‐79/CBP cells showed higher IC50 of CBP (Figure S1A), increased cell proliferation capability (Figure S1B), and reduced apoptosis (Figure S1C,D) compared with parental Y‐79 cells, indicating the successful establishment of the CBP‐resistant RB cell line. RT‐qPCR and western blotting results confirmed elevated mRNA and protein expression of USP49 in Y‐79/CBP cells compared to the parental Y‐79 cells (Figure 2A,B). Then, USP49 was overexpressed in Y‐79 cells and knocked down in Y‐79/CBP cells (Figure 2C,D). As shUSP49#2 showed better USP49 knockdown efficiency in Y‐79 cells, shUSP49#2 was applied for USP49 knockdown in the following experiments. As indicated by CCK‐8 results, the IC50 value for CBP in USP49‐overexpressed Y‐79 cells (41.36 μM) is significantly higher than in control (Vector‐transfected) Y‐79 cells (10.12 μM), with a *p* value likely <0.001; in addition, the IC50 value for CBP in Y‐79/CBP cells expressing (15.43 μM) is significantly lower than in control (shNC‐transfected) Y‐79/CBP cells (70.32 μM), with a *p* value likely <0.001 (Figure 2E). Furthermore, USP49 overexpression in Y‐79 cells increased cell proliferation and inhibited cell apoptosis, whereas the knockdown of USP49 in Y‐79/CBP cells exerted an opposite effect (Figure 2F–H). In sum, these results demonstrate that USP49 enhances malignant proliferation and facilitates CBP resistance of RB cells.

### 3.3 USP49 accelerates xenograft tumor growth and induces CBP resistance in vivo

**FIGURE 3:**
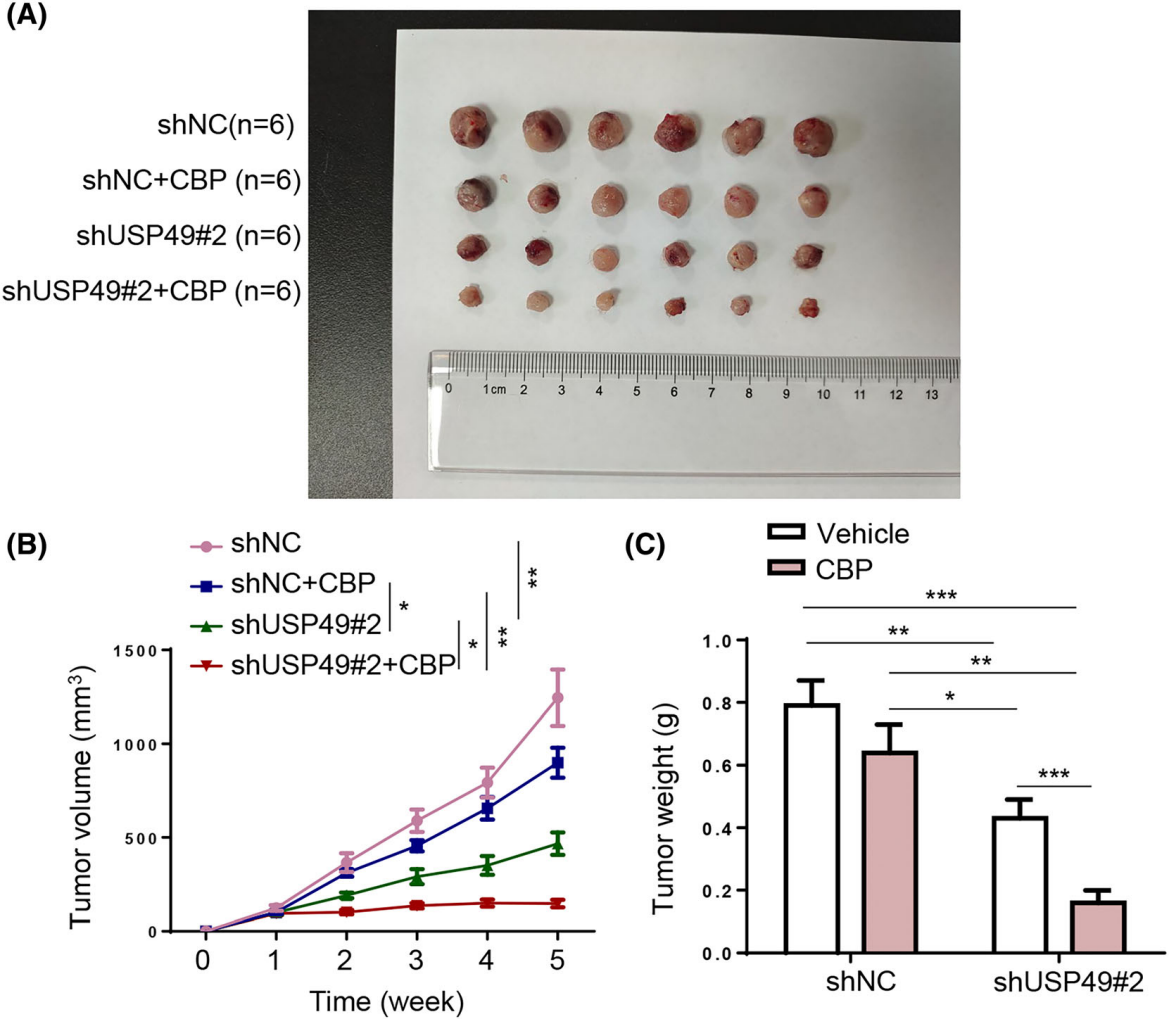
USP49 accelerates xenograft tumor growth and induces CBP resistance in vivo. (A) Images of xenografted tumors in nude mice bearing Y‐79/CBP cells from shNC, shNC+CBP, shUSP49#2, and shUSP49#2 + CBP groups (*n* = 6 mice/group). (B) The curves of tumor volume. (C) Tumor weight in each group. *p < 0.05; **p < 0.01.

Given the preceding in vitro results, murine RB xenografts were established to validate the oncogenic role of USP49 in RB in vivo. It was shown that USP49 silencing significantly attenuated xenograft tumor growth and impaired resistance to CBP (Figure 3A–C). Therefore, USP49 promotes RB tumor growth and CBP resistance in vivo.

### 3.4 USP49 promotes cell proliferation and CBP resistance in RB cells via autophagy activation

**FIGURE 4:**
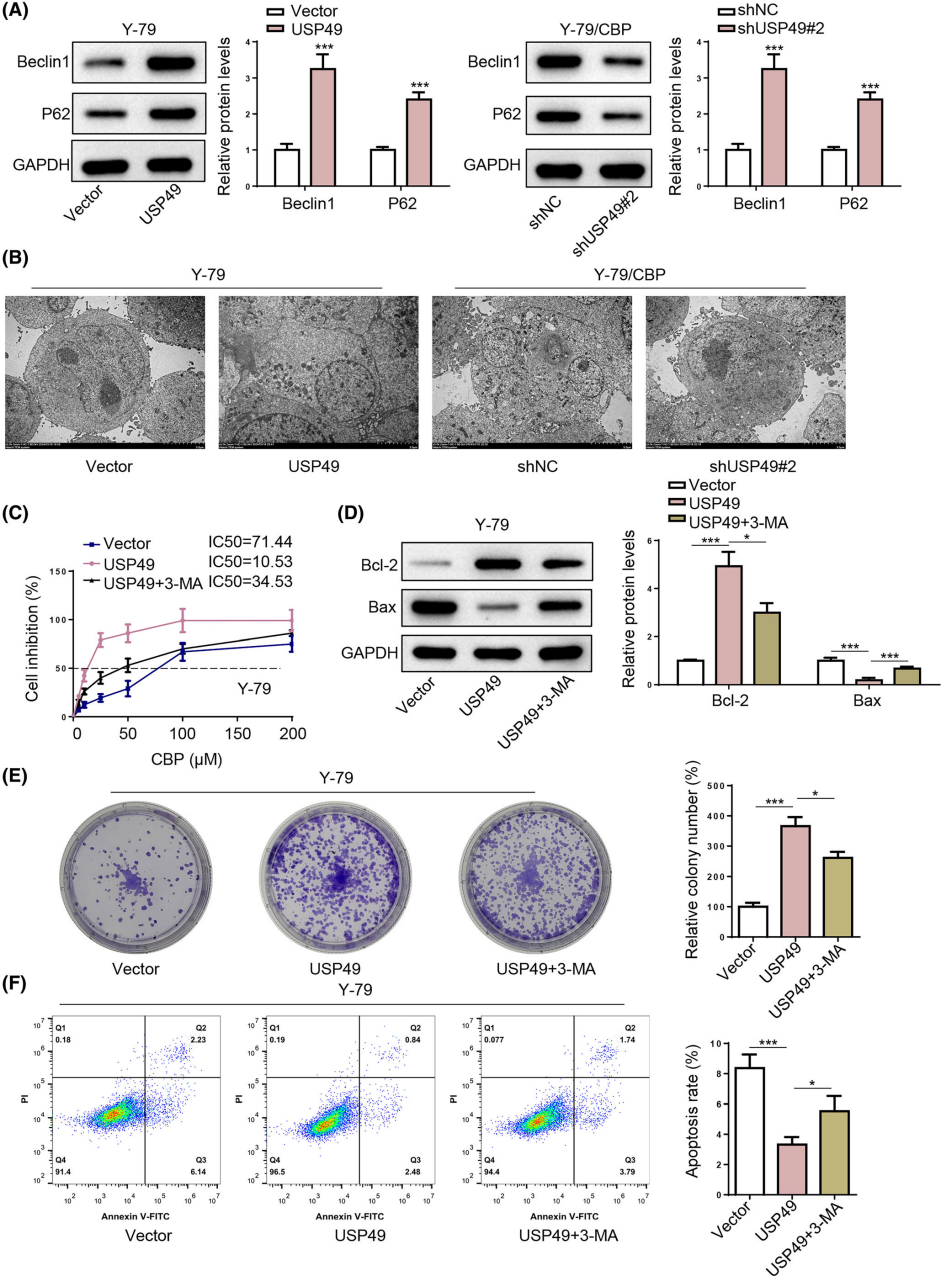
USP49 promotes cell proliferation and CBP resistance in RB cells via autophagy activation. (A) Beclin1 and P62 protein levels in Y‐79 cells and Y‐79/CBP cells from each group. (B) TEM images of autophagosomes in Y‐79 cells and Y‐79/CBP cells from each group. Then, Y‐79 cells were assigned to vector, USP49, and USP49 + 3‐MA groups. (C) IC50 value of Y‐79 cells from each group. (D) Cell proliferation of Y‐79 cells from each group. (E) The apoptotic rates of Y‐79 cells from each group. (F) Bax and bcl‐2 protein levels in Y‐79 cells from each group. *p < 0.05; **p < 0.01.

As autophagy activation is implicated in CBP resistance in RB,^15^ we explored whether USP49 might promote CBP resistance in RB by inducing autophagy. Western blotting results revealed that USP49 overexpression significantly increased Beclin1 expression and reduced P62 expression in Y‐79 cells, while USP49 deletion exerted opposite effects on Beclin1 and P62 expression in Y‐79/CBP cells (Figure 4A). Moreover, transmission electron microscopy (TEM) results showed a significant increase in autophagic vacuoles in USP49‐overexpressed Y‐79 cells and a significant decrease in USP49‐silenced Y‐79/CBP cells (Figure 4B). These results indicated that USP49 promotes autophagy activation in RB cells. Next, the autophagy inhibitor, 3‐methyladenine (3‐MA), was applied to block autophagy. The results showed that 3‐MA treatment reduced the IC50 value of CBP (Figure 4C), suppressed the colony‐formation capability (Figure 4D), and increased apoptosis (Figure 4E,F) in USP49‐overexpressed Y‐79 cells. Taken together, USP49 may confer CBP resistance in RB by promoting autophagy activation.

### 3.5 USP49 stabilizes SIRT1 by promoting SIRT1 deubiquitination

**FIGURE 5:**
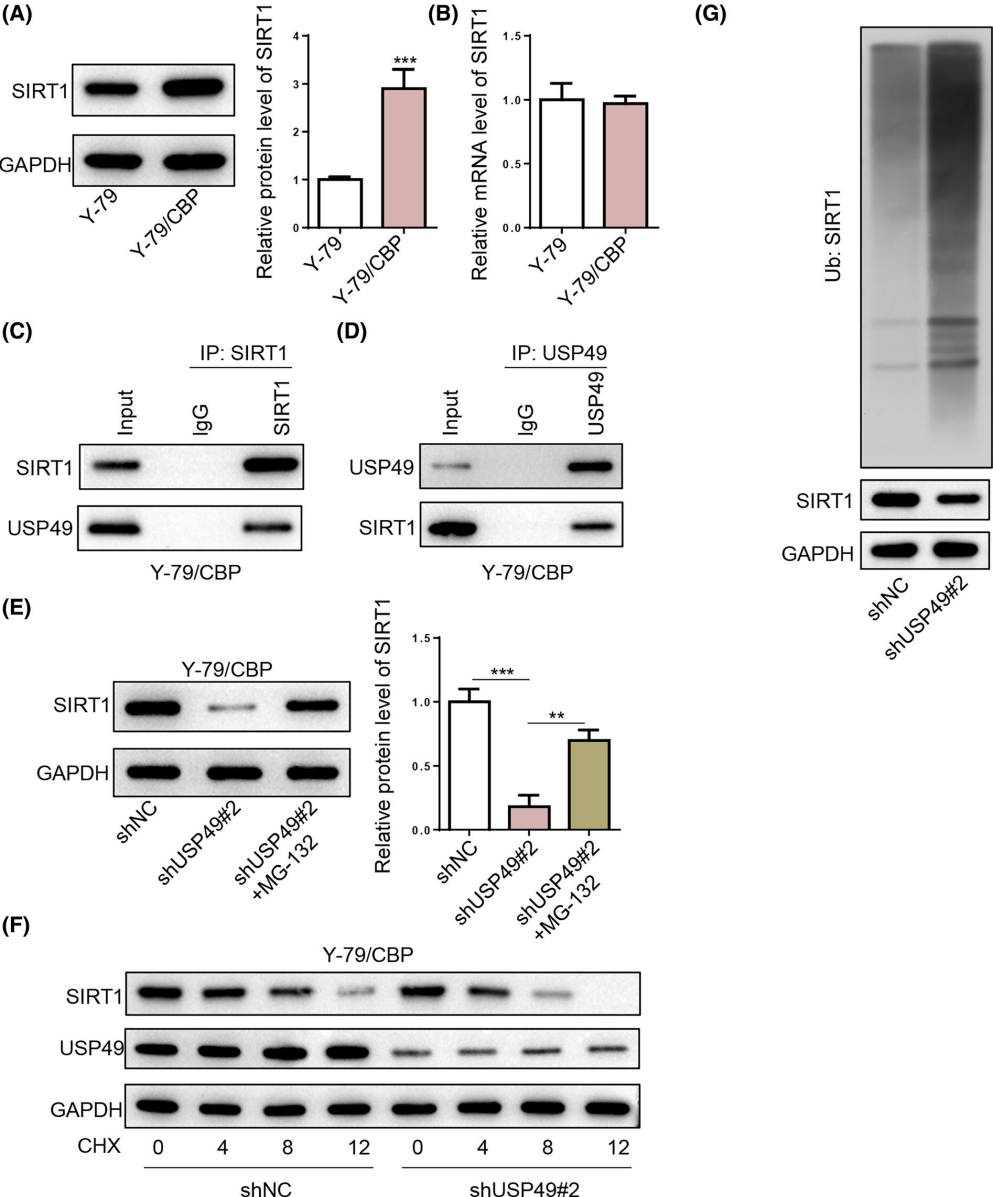
USP49 stabilizes SIRT1 by promoting SIRT1 deubiquitination. (A) SIRT1 protein expression levels in Y‐79 cells and Y‐79/CBP cells. (B) SIRT1 mRNA expression in Y‐79 cells and Y‐79/CBP cells. (C,D) The association between USP49 and SIRT1 was analyzed by Co‐IP. (E) SIRT1 protein expression level in Y‐79/CBP cells from shNC, shUSP49#2, and shUSP49#2 + MG‐132 groups. (F) The degradation profile of SIRT1 in Y‐79/CBP cells transfected with shNC or shUSP49#2 was assessed using the CHX chase experiment. (G) The ubiquitination levels of SIRT1 were determined by western blotting using an anti‐ubiquitin antibody. *p < 0.05; **p < 0.01.

SIRT1 has been reported to promote resistance to platinum‐based agents in human cancers by promoting autophagy.^24^ Interestingly, the SIRT1 protein expression level was remarkably increased in Y‐79/CBP cells compared with Y‐79 cells (Figure 5A), indicating that SIRT1 may participate in CBP resistance in RB. However, SIRT1 mRNA expression level remained nearly changed between Y‐79 and Y‐79/CBP cells (Figure 5B). As USP49 serves as a deubiquitinating enzyme,^25^ we speculated whether USP49 positively regulated SIRT1 protein level by protecting SIRT1 from proteasome‐dependent degradation. We first confirmed the interaction between USP49 and SIRT1 by Co‐IP. As shown in Figure 5C,D, USP49 co‐immunoprecipitated with SIRT1, suggesting an interaction between USP49 and SIRT1 in Y‐79/CBP cells. Impressively, USP49 silencing decreased SIRT1 protein level in Y‐79/CBP cells; moreover, the decrease in SIRT1 protein level could be reversed by the addition of proteasome inhibitor MG‐132 (Figure 5E), indicating SIRT1 may be degraded in a ubiquitin‐proteasome manner. In addition, the CHX chase experiment showed that the half‐life of SIRT1 protein was shortened after USP49 deletion in Y‐79/CBP cells (Figure 5F). Furthermore, USP49 knockdown remarkably increased the ubiquitination level of SIRT1 in Y‐79/CBP cells (Figure 5G). Taken together, these results suggested that USP49 interacted with SIRT1 and inhibited SIRT1 ubiquitination and degradation in RB.

### 3.6 SIRT1 overexpression reverses the effects of USP49 silencing in vitro

**FIGURE 6:**
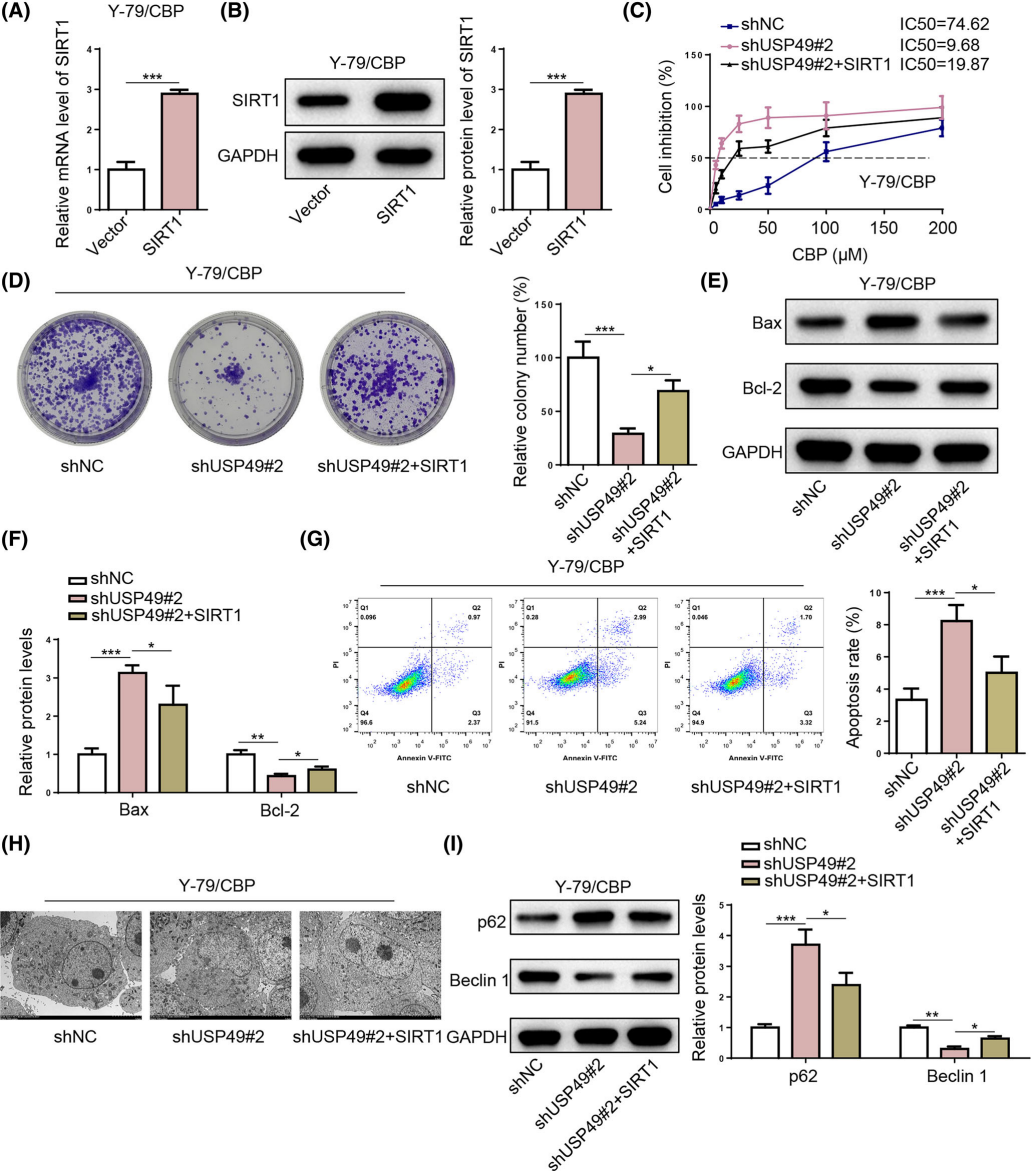
SIRT1 overexpression reverses the effects of USP49 silencing in vitro. (A,B) SIRT1 mRNA and protein expression levels in Y‐79/CBP cells transfected with vector or SIRT1. (C‐I) IC50 values of CBP, cell proliferation, apoptosis, and autophagy in Y‐79/CBP cells from shNC, shUSP49#2, and shUSP49#2 + SIRT1 groups. *p < 0.05; **p < 0.01.

To elucidate the biological functions of SIRT1 in USP49‐mediated CBP resistance in RB, we performed rescue experiments through simultaneous USP49 inhibition and SIRT1 overexpression in RB cells. The SIRT1 was overexpressed in Y‐79/CBP cells, with efficacy confirmed by RT‐qPCR and western blotting (Figure 6A,B). The experimental results showed that the effects of USP49 silencing on CBP resistance (Figure 6C), proliferation (Figure 6D), apoptosis (Figure 6E–G), and autophagy (Figure 6H,I) were largely rescued by SIRT1 overexpression. Altogether, SIRT1 is required for USP49‐mediated malignant proliferation and CBP resistance in RB in vitro.

### 3.7 IGF2BP3 upregulates USP49 expression in an m^6^A‐dependent manner

**FIGURE 7:**
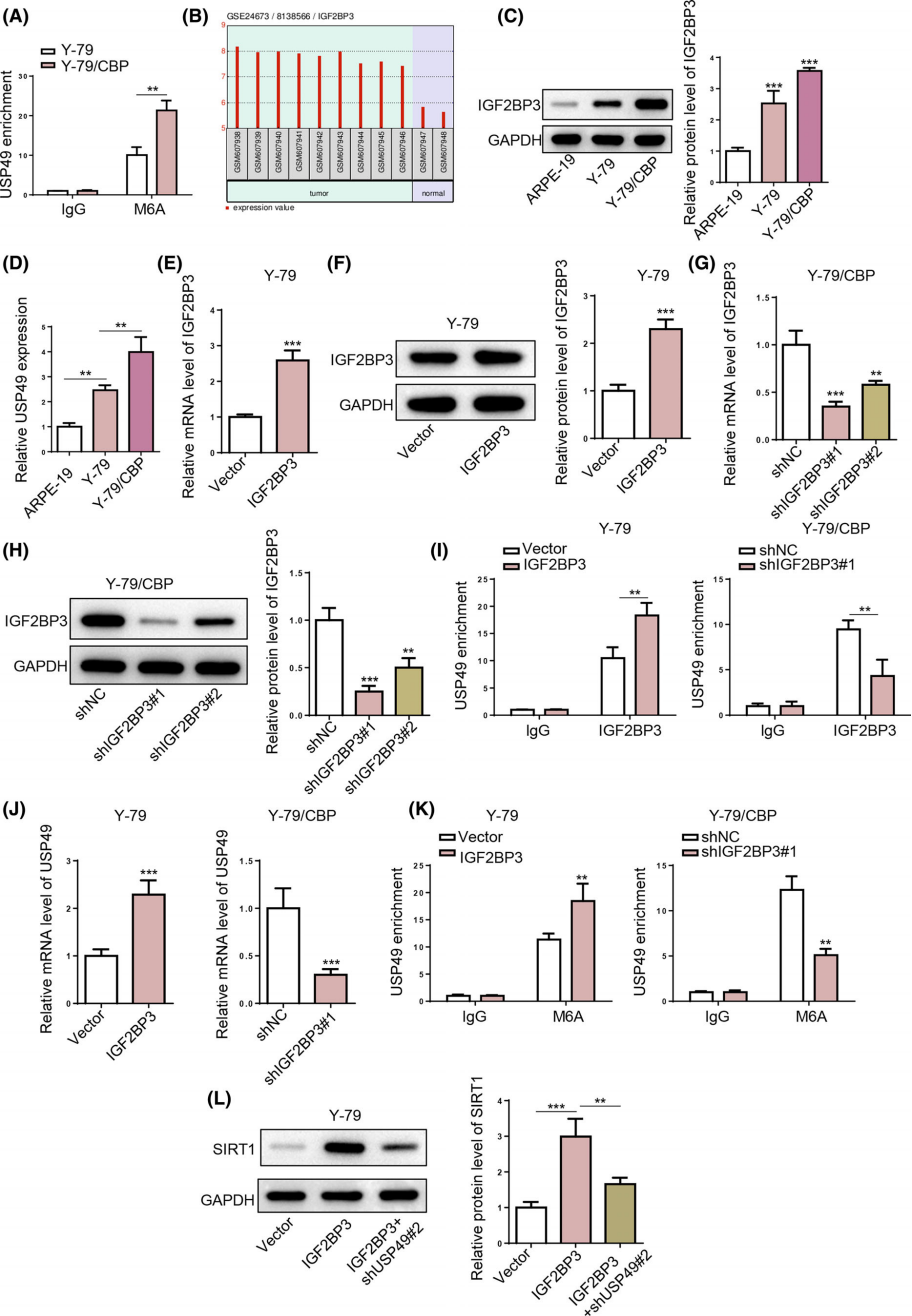
IGF2BP3 upregulates USP49 expression in an m^6^A‐dependent manner. (A) The enrichment of USP49 m^6^A in Y‐79 and Y‐79/CBP cells was detected by MeRIP‐qPCR analysis. (B) IGF2BP3 expression in RB tissues compared with normal tissues. (C,D) IGF2BP3 mRNA and protein expression levels in ARPE‐19, Y‐79, and Y‐79/CBP cells. Then, Y‐79 cells were transfected with Vector or IGF2BP3; Y‐79/CBP cells were transfected with shNC, shIGF2BP3#1, or shIGF2BP3#2. (E–H) IGF2BP3 mRNA and protein expression levels in Y‐79 cells and Y‐79/CBP cells. (I) The binding between IGF2BP3 and USP49 mRNA in Y‐79 cells and Y‐79/CBP cells was determined by RIP‐qPCR assay. (J) USP49 mRNA expression levels in Y‐79 cells and Y‐79/CBP cells from each group. (K) The enrichment of USP49 m^6^A in Y‐79 and Y‐79/CBP cells. (L) SIRT1 protein level in Y‐79 cells from Control, IGF2BP3, and IGF2BP3 + shUSP49#2 groups. *p < 0.05; **p < 0.01.

Previous research has shown that m^6^A methylation plays a pivotal role in retinal diseases, including RB.^26^ Since USP49 was upregulated in RB at both mRNA and protein levels, USP49 expression might be regulated via m^6^A modification. MeRIP‐qPCR analysis showed that the m^6^A level of USP49 mRNA was increased in Y‐79/CBP cells compared with that in Y‐79 cells (Figure 7A), indicating m^6^A modification might be involved in USP49 upregulation in RB resistance to CBP. As an m^6^A reader, IGF2BP3 has been reported to promote RNA stability in an m^6^A‐dependent manner.^27^ IGF2BP3 is associated with the resistance to platinum‐based agents in malignancies.^28^ Interestingly, IGF2BP3 was highly expressed in RB tissues (Figure 7B). In addition, IGF2BP3 expression was much higher in Y‐79/CBP cells, compared with Y‐79 and ARPE‐19 cells (Figure 7C,D). Furthermore, the online tool RM2Target (http://rm2target.canceromics.org/) predicted a binding relationship between IGF2BP3 and USP49. Then, IGF2BP3 was overexpressed in Y‐79 cells and silenced in Y‐79/CBP cells (Figure 7E–H). Since shIGF2BP3#1 exhibited better efficiency in knocking down IGF2BP3 expression in Y‐79/CBP cells than shIGF2BP3#2, shIGF2BP3#1 was applied for subsequent experiments. RIP‐qPCR assay showed that the IGF2BP3 specific antibody (IP) was significantly enriched with USP49 mRNA (Figure 7I), confirming the interaction between IGF2BP3 and USP49. It was shown that IGF2BP3 overexpression significantly increased USP49 mRNA levels; conversely, IGF2BP3 deficiency exerted the opposite effects (Figure 7J). Besides, Me‐RIP analysis demonstrated that IGF2BP3 overexpression significantly enhanced the m^6^A methylation level of USP49, whereas IGF2BP3 knockdown reduced the m^6^A methylation of USP49 (Figure 7K). We further observed that IGF2BP3 overexpression enhanced SIRT1 protein level which was reversed by USP49 knockdown (Figure 7L). Altogether, our findings indicate that IGF2BP3 upregulates SIRT1 by promoting m^6^A modification of USP49.

## 4 DISCUSSION

RB is the most prevalent pediatric intraocular tumor.^29^ The emergence of resistance to conventional chemotherapeutic agents poses a significant challenge in RB treatment, necessitating a deeper understanding of the underlying molecular mechanisms. In this study, we verified that IGF2BP3‐dependent m^6^A modification of USP49 promoted aggressive proliferation, inhibited apoptosis, and aggravated CBP resistance in RB by enhancing autophagy via SIRT1 deubiquitination.

USP49 has been reported as an oncogene in several cancer types. For instance, USP49 promotes the proliferation, metastasis, chemoresistance, and peritoneal metastasis in gastric cancer cells.^20^ Furthermore, USP49 is upregulated in adenocarcinoma of the esophagogastric junction and promotes cancer cell proliferation.^30^ In this study, bioinformatic analyses of the GSE24673 dataset identified significant upregulation of USP49 in RB tissues compared with normal tissues. Supporting this result, we also confirmed USP49 upregulation in RB cells. Interestingly, USP49 expression was elevated in CBP‐resistant RB cells compared to parental RB cells. Therefore, USP49 could potentially serve as a specific oncogene involved in RB progression and CBP resistance. Overexpression of USP49 promoted cell proliferation, inhibited cell apoptosis, and increased the IC50 value of CBP in RB cells. On the contrary, USP49 knockdown suppressed cell proliferation, increased cell apoptosis, and reduced the IC50 value of CBP in CBP‐resistant RB cells. Moreover, USP49 knockdown also suppressed tumor and CBP resistance in vivo. In sum, we suggest that USP49 confers aggressive proliferation and CBP resistance in RB, highlighting its potential as a therapeutic target.

Dysregulated autophagy has been implicated in various aspects of cancer progression, including chemoresistance.^31^ Inhibiting autophagy is still a traditional strategy to overcome CBP resistance in RB,^15^ and our study demonstrates that USP49 modulates key autophagy‐related proteins to enhance autophagy, thereby promoting RB cell survival and conferring CBP resistance. Furthermore, we provide insights into the underlying mechanism by which USP49 mediates CBP resistance in RB through autophagy activation. As a deubiquitination enzyme, USP49 also plays an essential role in cancer development and chemoresistance by regulating the protein stability of oncogenes or tumor suppressors.^20^ For instance, USP49 deubiquitinates and stabilizes Bcl‐2‐Associated Athanogene 2 (BAG2) which is a well‐known oncogenic protein that antagonizes apoptosis and enables adaptive response in colorectal cancer.^19^ In contrast, USP49 was discovered as a deubiquitinase of FKBP51, which is a tumor‐suppressing protein in pancreatic cancer.^32^ Herein, we identify USP49 as a novel SIRT1 regulator that stabilizes SIRT1 by inhibiting its ubiquitination.

SIRT1, an NAD^+^‐dependent histone deacetylase, has been shown to modulate multiple cellular processes, including autophagy, DNA repair, and apoptosis, thereby influencing the response to chemotherapy.^33^ Interestingly, recent studies have identified potential crosstalk between SIRT1 and other signaling pathways involved in chemoresistance, such as the PI3K/AKT pathway,^34^ the SDF‐1α‐CXCR4 signaling,^35^ and the NF‐ĸβ signaling pathway.^36^ Here, SIRT1 overexpression restored SIRT1 expression in USP49‐silenced CBP‐resistant RB cells and reversed the inhibitory effects of USP49 knockdown on CBP resistance, cell proliferation, and autophagy. Hence, USP49 may confer CBP resistance in RB by promoting SIRT1‐dependent autophagy activation.

Increasing evidence has indicated that dysregulated RNA m^6^A modification plays a critical role in the tumorigenesis and progression of human cancers.^37^ IGF2BP3, a member of the IGF2BPs family, is an m^6^A “reader” protein that selectively recognizes m^6^A‐containing transcripts to promote m^6^A‐mRNA degradation.^38^ Recent research has addressed the vital part of IGF2BP3 in carcinogenesis.^39^, ^40^ However, its role in RB and CBP resistance remains unclear. Our data identified that IGF2BP3 expression was upregulated in RB tissues and cell lines. Interestingly, RM2Target also predicted a binding relationship between IGF2BP3 and USP49. We thus speculated that IGF2BP3 participated in the m^6^A modification of USP49. By using MeRIP‐qPCR and RIP‐qPCR assays, we demonstrated that IGF2BP3 upregulated USP49 expression by enhancing the m^6^A level of USP49. In addition, IGF2BP3 overexpression enhanced SIRT1 protein level which was reversed by USP49 knockdown. Our findings revealed that IGF2BP3‐dependent m^6^A modification of USP49 mRNA upregulates USP49 expression to enhance SIRT1 stability.

These findings highlight the critical role of USP49 in cellular processes and its potential as a therapeutic target in RB. However, USP49 plays a crucial role in various cellular processes through its interactions with multiple proteins and signaling pathways. Consequently, targeting USP49 in clinical therapies may inadvertently impact other signaling pathways, resulting in unpredictable and potentially harmful outcomes. Future research should focus on developing inhibitors that selectively target USP49 in cancer cells. In addition, combining these USP49 inhibitors with agents that either protect normal tissues or enhance treatment specificity may help mitigate side effects, making such treatments more viable.

## 5 CONCLUSION

In summary, our study highlights the potential therapeutic implications of targeting USP49 and its associated pathways to overcome CBP resistance in RB. The study demonstrates that USP49 plays a critical role in promoting CBP resistance in RB. Furthermore, it was also revealed that IGF2BP3‐dependent m6A modification of USP49 enhances autophagy by stabilizing SIRT1 via its deubiquitination, helping RB cells survive under CBP treatment. Therefore, dual targeting of USP49 and autophagy pathways could offer a more effective therapeutic approach for RB management. By elucidating the molecular mechanisms driving resistance, we provide a rationale for the development of novel therapeutic strategies aimed at disrupting these pathways and improving treatment outcomes for RB patients.

## CONFLICT OF INTEREST STATEMENT

All authors declare no conflict of interest.

## Supporting information


**Figure S1.** Identification of Y‐79/CBP cell line. (A) IC50 of CBP for Y‐79 cells and Y‐79/CBP cells was detected using CCK‐8 assay. (B) Cell viability of Y‐79 cells and Y‐79/CBP cells was detected by CCK‐8 assay. (C) The apoptotic rates of Y‐79 cells and Y‐79/CBP cells were detected by flow cytometry. (D) Bax and bcl‐2 protein levels in Y‐79 cells and Y‐79/CBP cells. *p < 0.05, **p < 0.01.
