# Correction to “IGF2BP3‐Dependent N6‐Methyladenosine Modification of USP49 Promotes Carboplatin Resistance in Retinoblastoma by Enhancing Autophagy via Regulating the Stabilization of SIRT1”

**DOI:** 10.1002/kjm2.70289

**Published:** 2026-09-09

L. Li , N. Yang , J.‐H. Sun , L.‐J. Wei , and Y. Gao , “IGF2BP3‐Dependent N6‐Methyladenosine Modification of USP49 Promotes Carboplatin Resistance in Retinoblastoma by Enhancing Autophagy via Regulating the Stabilization of SIRT1,” Kaohsiung Journal of Medical Sciences 40, no. 12 (2024): 1043–1056, 10.1002/kjm2.12902.PMC1161849439497328

In the 2.10 Animal study paragraph of Section 2 Materials and Methods, the sentence “The present animal study was approved by the Ethics Committee of The Affiliated Hospital of Qingdao University” was incorrect. It should read: “The present animal study was approved by the Ethics Committee of The Hainan General Hospital (Med. Ethics Res. [2022] No. 262)”.

We apologize for this error.

