# Supplementary figures and images for: IGF2BP3‐dependent N6‐methyladenosine modification of USP49 promotes carboplatin resistance in retinoblastoma by enhancing autophagy via regulating the stabilization of SIRT1
Source: Kaohsiung J Med Sci. 2024 Nov 4;40(12):1043–56. doi: 10.1002/kjm2.12902 (PMC11618494; doi:10.1002/kjm2.12902)

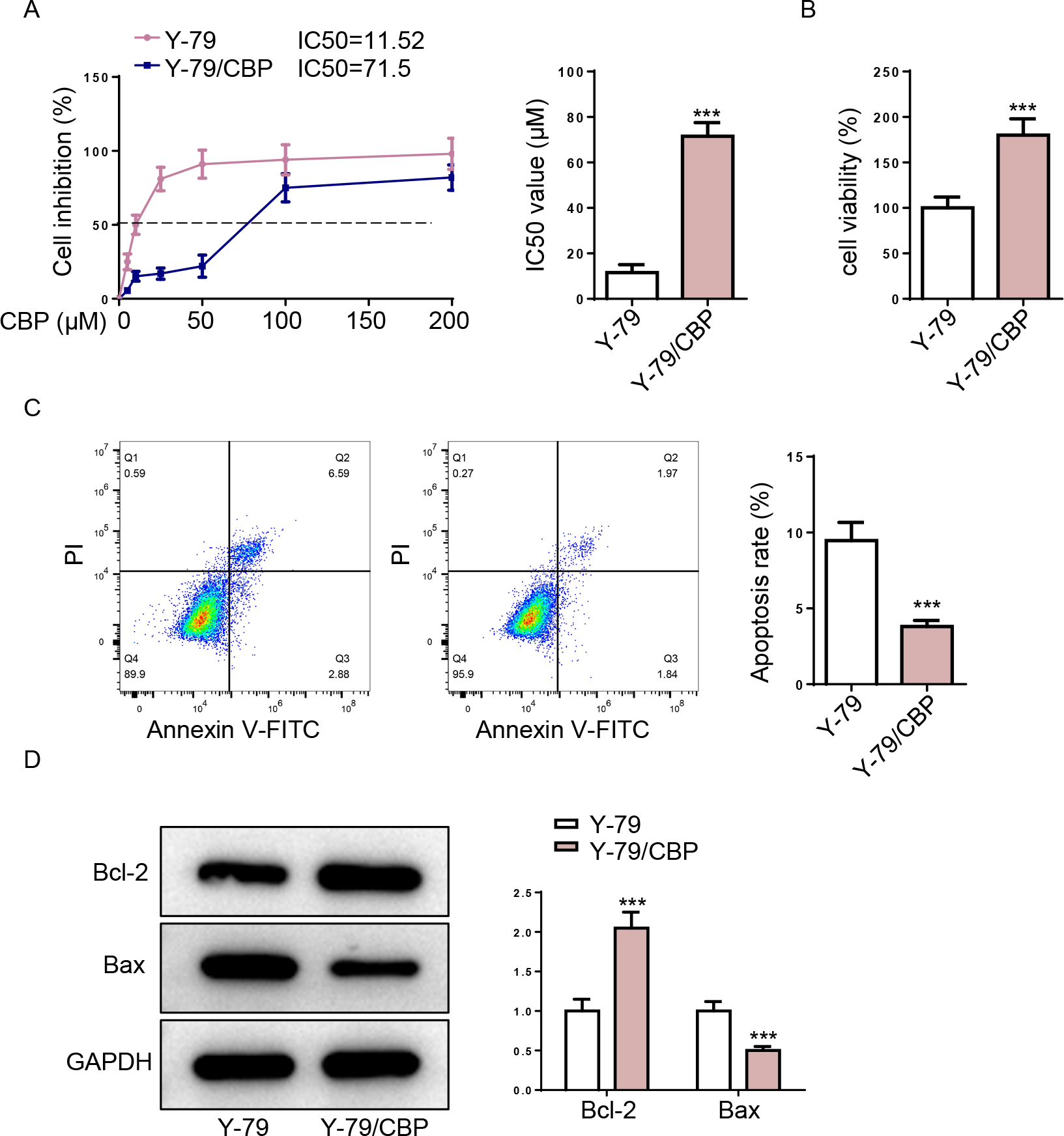

Supplement: Supplementary file 1 — Figure S1. Identification of Y‐79/CBP cell line. (A) IC50 of CBP for Y‐79 cells and Y‐79/CBP cells was detected using CCK‐8 assay. (B) Cell viability of Y‐79 cells and Y‐79/CBP cells was detected by CCK‐8 assay. (C) The apoptotic rates of Y‐79 cells and Y‐79/CBP cells were detected by flow cytometry. (D) Bax and bcl‐2 protein levels in Y‐79 cells and Y‐79/CBP cells. *p < 0.05, **p < 0.01. [file KJM2-40-1043-s001.tif]
